# Hydrogels for Engineering of Perfusable Vascular Networks

**DOI:** 10.3390/ijms160715997

**Published:** 2015-07-14

**Authors:** Juan Liu, Huaiyuan Zheng, Patrina S. P. Poh, Hans-Günther Machens, Arndt F. Schilling

**Affiliations:** 1 Department of Plastic Surgery and Hand Surgery, Klinikum Rechts der Isar, Technische Universität München, D-81675 Munich, Germany; E-Mails: liujuan_1018@126.com (J.L.); zhenghuaiyuan@126.com (H.Z.); patrina.poh@tum.de (P.S.P.P.); machens@lrz.tum.de (H.-G.M.); 2 Department of Hand Surgery, Wuhan Union Hospital, Tongji Medical College, Huazhong University of Science and Technology, Wuhan 430022, China; 3 Center for Applied Tissue Engineering and Regenerative Medicine (CANTER), Munich University of Applied Science, D-80335 Munich, Germany

**Keywords:** hydrogels, vascular networks, tissue engineering, fabrication

## Abstract

Hydrogels are commonly used biomaterials for tissue engineering. With their high-water content, good biocompatibility and biodegradability they resemble the natural extracellular environment and have been widely used as scaffolds for 3D cell culture and studies of cell biology. The possible size of such hydrogel constructs with embedded cells is limited by the cellular demand for oxygen and nutrients. For the fabrication of large and complex tissue constructs, vascular structures become necessary within the hydrogels to supply the encapsulated cells. In this review, we discuss the types of hydrogels that are currently used for the fabrication of constructs with embedded vascular networks, the key properties of hydrogels needed for this purpose and current techniques to engineer perfusable vascular structures into these hydrogels. We then discuss directions for future research aimed at engineering of vascularized tissue for implantation.

## 1. Introduction

The fabrication of three-dimensional (3D) constructs for cell culture and tissue regeneration is one of the major challenges for tissue engineering [1]. In the human body, cells are usually found within a range of 100–200 μm from the next capillary that provides the cells with oxygen and nutrients [2]. Lack of vascular networks in engineered tissues thicker than 200 μm therefore puts a limitation on their cells nutrition. Indeed, bigger 3D engineered tissues with densely encapsulated cells develop a necrotic core [3]. Advances in the engineering of ever more complex tissue constructs *in vitro* in recent years have therefore generated a necessity for a parallel development and design of 3D vascular networks [1,4]. Consequently, fabrication of perfusable vascular networks has become one of the most critical challenges in the advancement of tissue engineering.

To mimic the natural microenvironment of cells, a variety of 3D culture systems have been established. In most of these methods, hydrogels are used for embedding of the cells as they offer certain favourable properties like biocompatibility, similarity to components of the extracellular matrix (ECM) and adjustable chemical and physical characteristics. Consequently, hydrogels as biomaterials have also been widely used to engineer perfusable vascular scaffolds [5,6,7].

In this review, we first discuss different types of hydrogels used for fabrication of vascular network constructs and the properties needed for 3D casting and cell culture. Then we review different approaches to engineer vascular network constructs with hydrogels. Finally, we make conclusions and highlight the future challenges for hydrogel-based vascular engineering.

## 2. Hydrogels Used as Materials for Vascular Network Fabrication

Hydrogels are considered as an ideal class of materials for the engineering of soft tissue as they resemble some of the tissue properties better than other biomaterials. They have a similar high water content and can be manufactured to exhibit a good biocompatibility [8,9,10,11]. Hydrogels generally provide a suitable environment for cell growth with good exchange of oxygen and nutrients, as well as metabolic substances and cellular waste within the scaffolds [12,13,14,15]. Hydrogels with embedded vascular networks would closely mimic a natural extracellular environment or tissue. The development of such systems would therefore greatly promote complex studies of physiology and pathology, reduce the number of necessary animal experiments and may eventually lead to the development of engineered vascularized tissues. Different types of polymers with beneficial properties for this endeavor have been tested and are reviewed below.

### 2.1. Natural Hydrogels

Natural hydrogels consist of proteins, polysaccharides and protein/polysaccharide hybrid polymers. Especially collagen, gelatin and fibrin are commonly used proteins for vascular engineering.

Collagen type I has been widely used for cell encapsulation as well as for the study of cell traction. It has also been used to fabricate perfused microfluidic constructs *in vitro*. Kenneth *et al.* created three dimensional microvascular tubes within collagen gels. They built collagen tubes with a uniform diameter of 116 um, which did not change after five days of perfusion. Endothelialized tubes within the collagen exhibited microvascular properties. They possessed a strong barrier function, did not support the adhesion of leukocytes and responded to inflammatory mediators [16]. Based on the same material and technique, Wong *et al.* fabricated microfluidic collagen gels to study the *in vitro* effect of cAMP on the function and stability of microvessels [17]. Furthermore, collagen is considered an excellent biomaterial for artificial blood vessel development because of its integrin-binding sequences and low induction of inflammatory response [18].

Gelatin is the irreversibly hydrolyzed product of collagen. It retains some cell-binding motifs, such as RGD (Arg-Gly-Asp) and matrix metalloproteinase-sensitive degradation sites so that it has already become a commonly used biomaterial for vascular network engineering [19]. Paguirigan *et al.* fabricated a microfluidic device suitable for adherent cell culture and analysis by crosslinking gelatin with the naturally occurring enzyme transglutaminase. The study showed that post-crosslinked gelatin has a good cytocompatibility for murine mammary epithelial cells and fluidic access to cells was maintained during cell proliferation [20].

Fibrin is another biocompatible and elastic protein used for vascular network fabrication. The formation of fibrin is induced by addition of thrombin to solutions containing fibrinogen, which polymerizes into a fibrillar mesh. It can bind to critical proteins such as fibronectin, vitronectin, thrombospondin and vascular endothelial growth factor (VEGF) and has been used for cell-laden microfluidic devices and vascular engineering [21]. Xu *et al.* introduced a quasi-3D microfluidic model with parallel biocompatible fibrin hurdles mixed with other hydrogels. The models could be well-preserved in DMEM at 37 °C for more than two weeks. Cells were attached to the microfluidic channels and grew well. Cell behavior was studied on the hydrogel constructs [22]. Swartz *et al.* developed a fibrin-based vascular graft by incorporation of smooth muscle cells (SMCs) and endothelial cells (ECs) into the hydrogels, and implanted the graft into the jugular veins of lambs. They reported mechanical properties of the constructs comparable to native coronary arteries. Furthermore, more collagen and ECM were produced by SMCs in fibrin gels than in collagen gels [23].

Polysaccharides (e.g., hyaluronic acid (HA), agarose, alginate, chitosan) are another class of natural hydrogels that have been explored for the fabrication of constructs with microchannels, because of their good biocompatibility, good biodegradability, and excellent gel-forming properties [24,25,26]. The polysaccharides can be gelled through covalent crosslinking, polymerization, chemical conjugation, and esterification. Ling *et al.* molded microfluidic channels (50 × 70 μm and 1 mm × 100 μm) into agarose hydrogels embedded with cells. The study demonstrated that the hydrogels with microfluidic channels enabled efficient delivery of nutrients to the encapsulated cells [27]. Choi *et al.* created microfluidic channels (100 μm) in calcium alginate for the detection of mass transfer as well as quantitative control of the chemical environment surrounding encapsulated cells [28].

Sometimes, combinations of proteins and polysaccharides can form composite hydrogels such as collagen/Ha hybrid polymers, gelatin/chitosan and fibrin/alginate polymers [29,30,31]. Cuchiara *et al.* composed a collagen-HA semi-interpenetrating network hydrogel embedded with microfluidic structures [32].

The main limitations of hydrogels derived from natural polymers are their poor mechanical properties, which are generally difficult to control and tune, and their potential to induce immunogenic reactions [33,34,35]. In order to solve these problems, synthetic polymers have been explored and newly developed for this purpose.

### 2.2. Synthetic Hydrogels

Synthetic polymers have better reproducible physical and chemical properties than natural hydrogels. Previous vascularized constructs based on microfluidic systems were mainly built by using synthetic polymers including polydimethylsiloxane (PDMS), polyethylene glycol (PEG), poly(lactic-*co*-glycolic acid) (PLGA) and polyglycerol sebacate (PGS) polymers [36,37,38,39].

Although PDMS is not cytotoxic, cells cannot be cultured within PDMS, but only on the surfaces of PDMS or along the inner walls of channels, because it is not permeable to water. PLGA and PGS have been used to fabricate complex structures with microchannels on which endothelial and hepatocyte cells were seeded [40]. Their application was limited due to the relatively high volume of scaffold materials which needed to be degraded, and difficulties in achieving uniform cell seeding in the scaffold.

PEG is one of the most widely used synthetic hydrogels for tissue engineering. It is soluble in water and inorganic solvents such as methanol, ethanol, dichloromethane *etc.* It has a low protein adhesion and is non-immunogenic [41,42]. Liu *et al.* have introduced microfluidic PEG hydrogels to engineer hepatic tissue constructs [43]. In addition, PEG-based hydrogels can be synthesized by modifying end hydroxyl groups with functional groups (carboxyl, thiol and acrylate) or combining them with other molecules or bioactive agents [44]. For example, Kloxin *et al.* fabricated microchannels by photopatterning PEG-based hydrogels to investigate 3D cell behavior [45]. Sarig-Nadir *et al.* observed cell growth and connectivity along microchannels by laser photoablating PEGylated fibrinogen hydrogels [46].

Another class of synthetic hydrogels used for microfluidic fabrication are self-assembling peptide hydrogels. Due to their high biocompatibility and biodegradability, tailorable bioactive properties and the ability to be self-organized into nanofibrous architecture, which mimics the natural ECM, they have been introduced as possible biomaterials for tissue engineering applications [47]. There are two major types of self-assembling peptides: self-complementary peptides (SCP) and peptide amphiphiles (PAs). Yu *et al.* built PDMS-based microfluidic chips coated with ionic complementary peptide EAR16-II. It interacted strongly with the surface of PDMS in the same way as proteins and therefore minimized nonspecific protein adsorption and improved biocompatibility of these microfluidic chips [48]. Goktas *et al.* fabricated PAs and PEG composite hydrogels as tunable ECM mimetic microenvironment. The resulting porous network could be functionalized with desired bioactive signaling epitopes and the mechanical properties of the composite hydrogels could be precisely controlled [49].

### 2.3. Hybrid Hydrogels

As natural hydrogels exhibit better biocompatibility and cell affinity than synthetic hydrogels, but have poorer tailoring properties for mechanical strength, water content, and degradation rate, it seems to be an obvious solution to combine natural and synthetic hydrogels to capitalize on the advantages of both. As an example, PEG hydrogels have adjustable mechanical properties and allow easy control of the scaffold architecture. However, because of their bio-inert nature, they cannot provide a good environment for cell adhesion and tissue formation without any modification [44]. Fibrinogen is an important adhesive protein in the ECM which has several binding domains for cell membrane [50]. To integrate natural bioactive molecules into PEG hydrogels, PEGylated fibrinogen hydrogels were synthesized. They have been used to fabricate microfluidic constructs by laser photoablation for directing cell growth, and showed good mechanical properties and cell affinity [50]. Hybrid hydrogels with higher percentage of PEG (PEG/fibrinogen, 75:1) showed better maintenance of the microchannels than hydrogels with a lower ratio (PEG/fibrinogen, 45:1).

Another example of a hybrid approach for vascular tissue engineering is gelatin, electrospun together with poly(l-lactic acid), which formed homogenous vessel-like tubular scaffolds with favorable physical and biological properties [51].

It is also possible to synthetically modify natural polymers. Kageyama *et al.* integrated hydrazide-modified gelatin with aldehyde-modified HA as *in situ* cross-linkable hydrogels to rapidly fabricate perfusable vascular-like constructs [52]. Another example is Gelatin methacrylate (GelMA) which is synthesized by replacing the amine groups of gelatin with methacrylic functional groups, which makes it photopolymerizable [53]. It has been used to engineer cell-laden microfluidic devices and maintained better mechanical properties than gelatin [54]. GelMA was easily patterned down to 100 μm resolution with enough robustness. Cell adhesion, proliferation, elongation as well as migration were observed both when cells were seeded on the surface and when they were encapsulated within GelMA hydrogels.

## 3. Key Hydrogel Properties for Vascular Engineering

### 3.1. Mechanical Properties

**Figure 1:**
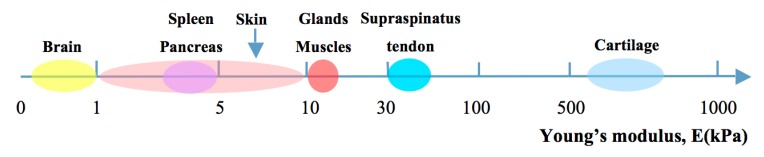
Young’s modulus of natural soft tissues and organs in kPa.

The most commonly investigated mechanical property of hydrogels for tissue engineering is their Young’s modulus, which is a geometry-independent measure of stiffness. For native soft tissues and organs, the Young’s modulus ranges from 0.1 kPa to 1 MPa (Figure 1) [55,56,57,58], depending on the function and location of the tissues. Ideally, the hydrogel scaffolds for vascular engineering should provide appropriate stiffness mimicking the proposed native ECM and allowing to be casted, support cell growth and maintain vascular architecture under perfusion for a period of time [59]. Ling *et al.* demonstrated that 3% agarose hydrogel with a reported stiffness of 19–32 kPa is rigid enough to support channels under perfusion without deformation. The stiffness of hydrogels can be varied by changing polymer concentration, physical or chemical crosslinking, increasing the degree of crosslinking, removing divalent metal ions and adjusting environmental conditions (e.g., pH value, temperature) [60,61]. Bryant *et al.* fabricated PEG-based hydrogels with Young’s moduli ranging from 60 to 500 kPa by increasing the concentration of polymer from 10% to 20% [62]. Huang *et al.* enhanced the Young’s modulus of HA-tyramine hydrogels from 5.4 to 11.8 kPa by increasing the degree of crosslinking, which was achieved by adjusting the concentration of hydrogen peroxide from 500 to 1000 μM [63].

Furthermore, mechanical properties of hydrogels play an important role for the cellular behavior. Human mesenchymal stem cells (hMSCs) exhibit neurogenic differentiation when cultured on hydrogels with stiffness similar to brain (0.1–1 kPa), but show myogenic and osteogenic phenotypes on hydrogels with similar stiffness as muscle (8–17 kPa) and nascent bone (>34 kPa), respectively [63]. The change of pore sizes in tissue-engineered constructs can also influence their mechanical properties. These are not only important for cell behavior, but also for diffusion of nutrients, mass transport and tissue formation [64]. The pore size furthermore influences cell migration, proliferation as well as vascularization *in vivo* [65]. The minimum pore size for non-vascularized tissue engineering constructs for regenerating mineralized bone is considered to be 100 μm [66]. Smaller pores (10–75 μm) were penetrated only by fibrous tissue and pore sizes >300 μm were recommended for bone tissue regeneration without engineered embedded vessels [67].

### 3.2. Gelation Time

The time duration for hydrogels to complete gelation is another important factor for the generation of perfusable vascular networks. Gelation needs to complete so the channel walls are strong enough to support the perfused gel. Consequently, gelation time of hydrogels needed for the fabrication of vascular network constructs depends on methods and purposes. When fabricated by molding, gelation should be as fast as possible to reduce oxygen and nutrient depletion. Especially encapsulation of organ-specific cells within hydrogels leads to restrictions in possible timing, as some cells can not sustain long time (>30 min) oxygen and nutrition depletion [5]. On the other hand for 3D bioprinting, excessively rapid and premature gelation will lead to an increase in the viscosity of the injectable solution, thereby interfering with the printing or even blockage of the needle [68].

The gelation time varies for different hydrogels. Gelation of collagen and matrigel usually takes more than 20 min at 37 °C, even if the hydrogels are only a few millimeters thick [69]. Gelatin needs more than 15 min at room temperature for gelation while it takes less than 5 min when crosslinked with mTG. Gelation time can be modulated by increasing concentration of crosslinking agents, or by introduction of chemical modifications. Kageyama chemically modified gelatin with hydrazide (gelatin-ADH) and HA with aldehyde (HA-CHO), and achieved rapid gelation by simply mixing solutions of these two components. In addition, gelation time is dependent on the concentration of combined hydrogels [52]. For example, in Kageyama’s study, the gelation time of G2.5H1 (concentration of gelatin-ADH and HA-CHO were 2.5% and 1% respectively) was 18 ± 3.0 s while for G7.5H3 was 3 ± 0.3 s.

### 3.3. Gelation Mechanisms

Hydrogels for vascular network fabrication are formed through gelation mechanisms where hydrogel precursors are crosslinked physically, chemically or through radical polymerization.

Physical crosslinking is usually induced by change of temperature, charge of solution, or pH value, which leads to entangled chains, hydrogen bonding, hydrophobic interaction and crystallite formation [70]). It solidifies the hydrogels and maintains their shapes in culture medium. In Ling *et al.*’s study, agarose solution was prepared by heating agarose in phosphate-buffered saline above 80 °C until dissolved. The agarose-cell mixture was gelled at 25 °C to form a stable microfluidic structure [27]. Self-assembled peptide gelation takes place when self-assembed peptide hydrogels are exposed to electrolyte solution or salt solution, where the net charge of the peptide molecule is near zero [14]. However, the crosslinking might be reversible and not permanent.

Chemical crosslinking is the process of binding two or more peptide molecules chemically by a covalent bond based on enzymatic reactions. Crosslinking reagents contain reactive end groups that respond to specific functional groups (e.g., primary amines, carboxyls and sulfhydryls). Transglutaminase is one of the chemical crosslinking reagents. It can mediate the chemical reaction between glutamine and lysine residues on adjacent protein fibers. The formed covalent amide bonds reinforce the 3D structure of the hydrogel. It is usually used to crosslink collagen and gelatin for their application in tissue engineering. Paguirigan *et al.* created microchannels using gelatin crosslinked with the naturally occuring enzyme transglutaminase and produced microfluidic devices for cell culture [20]. However, the gelation time is longer compared to physical crosslinking and radical polymerization.

For radical polymerization, hydrogels are modified by addtion of vinyl groups (e.g., methacrylate or fumarate) to form end-functionalized macromers [71]. High-molecular-weight chains formed by carbon-carbon double bonds in the hydrogel precursors are crosslinked in the presence of an initiator (e.g., light) [14]. For example: Addition of methacrylate groups to the amine-containing sides of gelatin (GelMA) make it polymerizable when exposed to light. Nichol *et al.* synthesized GelMA and created microfluidic hydrogel devices through radical polymerization. PEG-based hydrogels can also be prepared by radical polymerization used for vascular network fabrication [44]. However, sometimes the initiator may be toxic to cells, so that appropriate initiating conditions (e.g., initiating density, exposure time) have to be considered during hydrogel fabrication [72].

### 3.4. Cytocompatibility and Cell Attachment

Cytocompatibility is an essential requirement for engineering of cell laden constructs and has therefore been extensively studied, usually using live/dead fluorometric assays. Yung *et al.* encapsulated human embryonic kidney cells (HEK293) in mTG-crosslinked gelatin hydrogels and observed that the cells could maintain high viability for at least three weeks [73]. In agarose hydrogels with perfusable microchannels AML-12 murine hepatocytes showed good viability near the perfusion lumen [27].

Cell attachment is one of the most important hydrogel properties for perfusable vascular network constructs as it is a prerequisite for population of the channel walls by endothelial cells and important for cell behavior and function [74]. Cell attachment varied in different types of hydrogels as well as in different concentrations of the same hydrogel. Higher concentrations of gelatin were shown to lead to better cell attachment. Skardal *et al.* compared cell attachment and proliferation on hydrogels created by mixing different ratios of thiolated HA:thiolated gelatin. Cell attachment increased gradually along with the increase of gelatin ratios from 0% to 75%, with no significant difference of cell attachment between the ratios of gelatin 75% and 100% [75].

Cell attachment can also be modulated through increasing the number of cell attachment sites in hydrogels. One possible method for this is conjugating collagen-associated proteins (CAPs) chemically on the hydrogel network. Proteins with RGD (Arg-Gly-Asp) sequences are the most common CAPs used for cell-attachment modification. When seeding human umbilical vein endothelial cells (HUVECs) on PEGDA hydrogels, no spreading was observed 4 h after seeding and the cells possessed a round morphology. However the cells showed initial cell attachment and spreading 4 h after seeding on RGD incorporated PEGDA hydrogels and extensive spreading after 24 h. The increasing of cell attachment was attributed to the specific binding of HUVECs to the RGD ligands on the RGD incorporated PEGDA hydrogels [76].

### 3.5. Degradation

Degradation of hydrogels used for vascular network fabrication is important because the embedded cells need space to spead, grow, proliferate and migrate. That means once the hydrogel constructs are embedded with cells or placed at the application site, they should be able to bear the local mechanical loads and perfusion stress for a time period until the cells produce their own functional ECM. If the degradation is too fast, dissolution of the supporting hydrogel may lead to collaps of engineered vascular structures. If the degradation is too slow, cell proliferation and tissue formation might be retarded [72]. The degradation can also be used for a slow release process. This has been demonstrated in MSCs, which showed a round shape when encapsulated in PEG hydrogels. In the course of degradation of the surrounding hydrogel, the cells migrated to other cells, forming cell-cell junctions, and presented a more osteoblastic phenotype [13].

The primary mechanisms of degradation of hydrogels are through hydrolysis, enzyme-mediated, or a combination of both [14,77]. Synthetic hydrogels can be created with a desired degradation rate that matches tissue regeneration and growth. This can be achieved by incorporating hydrolytically or enzymatically labile segments [14]. Hydrogel degradation *in vivo* or *in vitro* is also affected by other environmental factors, including the presence of cells.

In summary, the properties of hydrogels required for vascular engineering depend on the cells encapsulated, the cells that act as endothelial wall and the mechanical properties of the tissue they mimic. For the fabrication of most multivascular constructs, ideal hydrogels should have a short gelation time to reduce the depletion of oxygen and nutrients in the fabrication step, a proper mechanical stiffness to maintain microchannels and sustain the perfusion, and good cell affinity. It should also provide ECM for encapsulated cells and allow cells to degrade the hydrogels and remodel their surrounding environment. The hydrogel precursors, gelation mechanisms as well as degradation products must be cytocompatible.

## 4. Methods for Vascular Network Scaffold Fabrication

### 4.1. Molding

**Figure 2:**
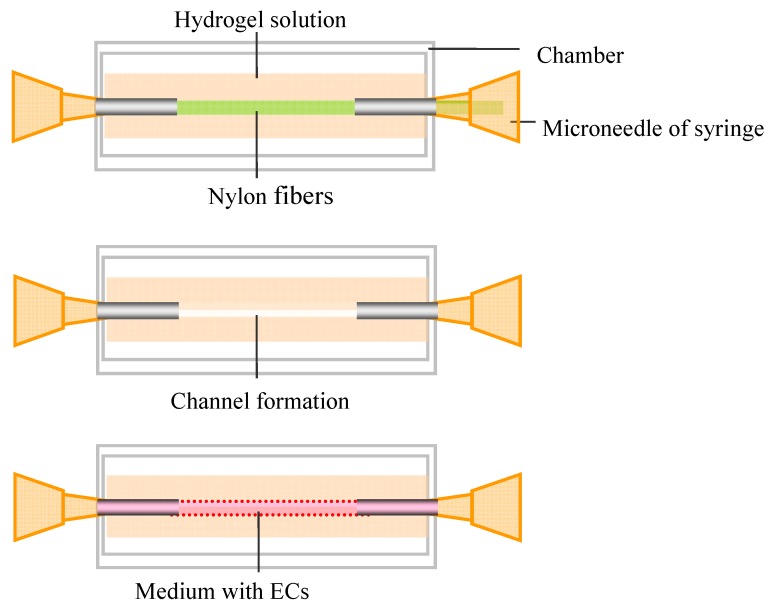
Molding approach for fabrication of microfluidic constructs. Microneedles and a Nylon fiber are fixed inside a chamber. Then hydrogel solutions are poured into the chamber and gelled. Then open channels are formed by gently removing the fibers. Medium with ECs can be perfused through the channel.

Molding is one of the most primitive and commonly used methods to fabricate hydrogel constructs containing perfusable vascular networks. Usually microneedles or fibers are fixed inside a chamber according to the designed patterns. Then hydrogel solutions are poured into the chamber and gelled. Then open channels are formed by gently removing the needles or fibers. With this technique, Takayuki *et al.* fabricated a perfusable single capillary-like network in type I collagen by using a tube (550 μm in diameter) seeded with endothelial cells (ECs) to mimic the capillary structures (Figure 2). They considered this approach as a starting point for the formation of a self-developing capillary-like network [7]. Chrobak *et al.* fabricated open channels (diameter: 100 μm) in collagen hydrogels [16]. ECs were seeded on the channel surface to form endothelialized tubes. They also found cellular organization and function similar to human microvessels in the tubes [78,79]. Nichol *et al.* used microneedles within GelMA hydrogels encapsulated with NIH 3T3 fibroblasts to form endothelialized tubes, which can be potentially used to co-culture different types of cells in the microvascularized tissue constructs.

Beside the normal molding to make vascular constructs, Kageyama *et al.* described an innovative approach to generate perfusable microchannels with *in situ* crosslinkable hydrazide-modified gelatin and aldehyde-modified HA where the surface of the channels was fully enveloped with HUVECs. They used zwitterionic oligopeptides as a self-assembled molecular layer on a gold needle, which allowed HUVECs to attach. Then the cells were transferred onto the inner surface of the hydrogel by electrochemical desorption of the oligopeptides. This group provided a very fast (gelation less than 20 s) and efficient method of fabricating perfusable vascular constructs with endothelial cells [52].

However, traditional molding methods with microneedles or fibers are mainly confined to fabrication of only simple architectures with straight and parallel microchannels.

### 4.2. Soft Lithographic Approach

**Figure 3:**
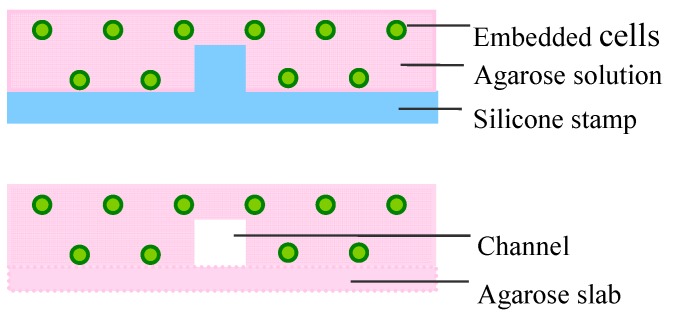
Soft lithographic approach for fabrication of microfluidic constructs. Agarose solution embedded with hepatic cells was gelled on a SU-8 patterned silicon wafer and microfluidic channels were formed by heating and sealing of the surface of two molded agarose hydrogels.

Soft lithographic approaches for fabricating microfluidic hydrogels were based on templates predefined with vascular patterns (e.g., silicone, PDMS). Zheng *et al.* engineered microfluidic vascular circuits via soft lithography in a type I collagen gel. The key steps are molding collagen on a microstructured silicone stamp using injection-molding techniques, which consist of *in situ* gelation of collagen on the silicone stamp and formation of the enclosed fluidic structure by sealing two collagen layers together. The structured hydrogel has an inlet and an outlet, which enable perfusion with medium as well as the seeding of ECs during 3D cell culture [6]. Ling *et al.* created microfluidic hydrogels through a similar approach. Agarose solution embedded with hepatic cells was gelled on a SU-8 patterned silicon wafer and microfluidic channels were formed by heating and sealing of the surface of two molded agarose hydrogels (Figure 3) [27]. In addition, microfluidic devices fabricated in PDMS have been introduced in the research on cardiovascular systems. Paguirigan *et al.* fabricated three positives in PDMS molds and microchannel constructs were generated with gelatin crosslinked by transglutaminase on a PDMS mold [80].

The soft lithographic approach is limited to creating 3D constructs with individual layers stacking to each other. The fabrication of hydrogel constructs containing vascular networks needs further steps for patterning and stacking.

### 4.3. Photopatterning Approach

**Figure 4:**
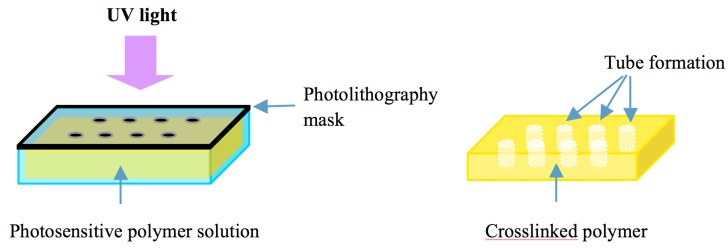
Photopatterning approach for fabrication of microfluidic constructs. A thin film of a photosensitive polymer is exposed to UV light through a predesigned mask. There are transparent regions in the mask that allow the UV light to pass through. The areas where the light can reach through the transparent regions generate crosslinking of the polymer while the dark areas under the mask that cannot receive the light will maintain liquid and can be easily removed to form tubular structures.

Methods based on photopatterning have been widely used for fabricating tiny tissue constructs with hydrogels which can be polymerized or crosslinked by exposure to light. In the photopatterning processes, a thin layer of a photosensitive polymer with photopolymerizable groups (e.g., methacrylate and acrylate) is exposed to UV light through a mask. There are transparent regions in the mask that allow the UV light to pass through. Crosslinking of photosensitive polymers under the transparent regions can be induced by activation of a photoreaction. This method is fast and highly reproducible and has been used to fabricate hydrogel constructs with predesigned microchannels for vascularization or perfusion (Figure 4). Liu *et al.* generated polygonal channels in cell-laden PEG hydrogels using a layered photopatterning approach [43]. Du *et al.* fabricated predefined microchannel units using a photolithography approach and assembled the units into a large tubular construct with multi-level interconnected lumens [81]. Alessandro *et al.* created vascularizable hydrogels with GelMA gelation under UV lamp based on PVA sacrificial templates [82].

Potential disadvantages of photocrosslinkable materials and UV light are toxic effects on cells. The depth of UV light penetration in hydrogels is limited, therefore the methods of fabricating thicker perfusable vascular network constructs might require further assembly to generate 3D tissues.

### 4.4. 3D Bioprinting

3D bioprinting is the process of printing 3D constructs using cells and biomaterials. Another description of 3D bioprinting is “layer-by-layer additive robotic biofabrication of three-dimensional functional living macrotissues and organ constructs” [83]. Compared to traditional 3D printing in the manufacturing industry, other materials are used. For 3D bioprinting usually phase-changing hydrogels without cytotoxic chemicals and gentle dispensing technology are needed. The printers can simultaneously deposit biomaterial scaffolds and cells at precisely controlled 3D-locations.

**Figure 5:**
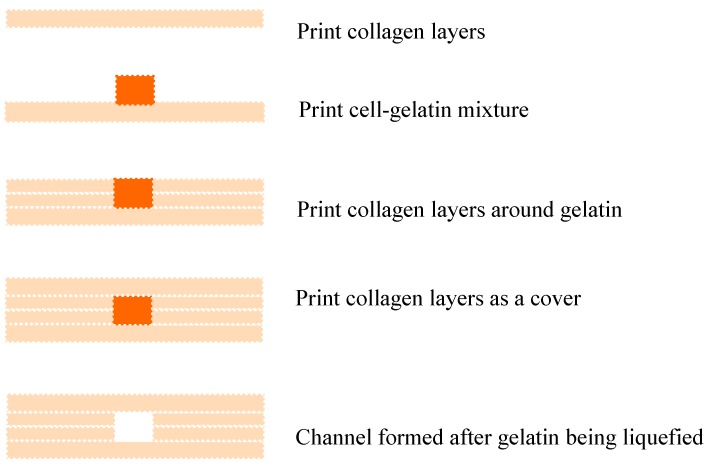
Bioprinting for fabrication of multi-level tubular constructs. With a layer-by-layer printing approach, the collagen precursor was printed and polymerized. Then gelatin/cell mixture was printed in a straight pattern to take the position of the channel. After gelation, more collagen layers were printed to cover the gelatin pattern. The vascular structure was formed after gelatin was liquefied.

Lee *et al.* developed a bioprinting approach to construct a perfusable channel in collagen scaffolds [84]. Collagen hydrogel precursor was used as a scaffold material for bioprinting. Gelatin was used as a sacrificial material to create channels. With a layer-by-layer approach, the collagen precursor was printed and polymerized by NaHCO_3_, then 20% gelatin solution embedded HUVECs were printed in a straight pattern to take the position of the channel. After solidification of gelatin, more collagen layers were printed to cover the gelatin pattern. The vascular structure was formed when collagen completed gelation and gelatin was liquefied (Figure 5). The channel maintained its structure integrity for two-weeks in tissue culture under flow conditions without leakage, flow turbulence or shrinking of the scaffold material.

Aleksander *et al.* introduced the open source Fab@Home printing system to bioprint vascular tissue constructs [85]. They generated printable hydrogels by in turn co-crosslinking of thiolated HA and gelatin derivatives with TetraPAcs. These modular synthetic hydrogels can be extruded as sausage-like cellularized macrofilaments and were used to bioprint vascular tissue constructs [86]. They also generated tubular constructs with extrudable gel-like fluids by co-crosslinking of GelMA with methacrylated hyaluronic acid (HAMA) [75].

### 4.5. Sacrificial Molding of Perfusable Channels

As reported, the molding approach for fabricating perfusable channels is limited to generate simple vascular structures and bioprinting has limitations in printing speed as well as materials and cells. To solve these problems, sacrificial molding provides an intriguing alternative [87,88]. For this, a template of microchannels is created through molding or bioprinting. It is then fixed in a chamber filled with hydrogel precursors. After complete gelation of hydrogels, it is sacrificed by self-degradation at 37 °C or dissolved in a special solvent to form a microchannel architecture.

Glass and polymer fibers are commonly used sacrificial materials in this approach. Nazhat *et al.* introduced soluble phosphate glass fibers in collagen scaffolds [89]. The glass fibers were degraded in water at 37 °C, and microchannels (Diameter: 10 to 50 μm) were formed within the collagen scaffold. Andrew *et al.* encapsulated gelatin meshes in collagen and fibrin. The gelatin meshes were melted and flushed at 37 °C, with interconnected channels left behind in the hydrogel [90]. Alessandro *et al.* fabricated perfusable hydrogel scaffolds using poly(vinyl alcohol) (PVA) as the sacrificial template. The PVA templates were molded with multi-microfluidic patterns and fixed in a bioreactor. After complete crosslinking of the hydrogels surrounding the PVA, the templates were removed by washing with water or PBS to form microfluidic gels. This method can also be used to fabricate multilayered microfluidic gels by stacking multiple sacrificial templates [87]. In addition, Madden incorporated polycarbonate core/poly(methyl methacrylate) (PMMA) optical fibers into poly(2-hydroxyethyl methacrylate-*co*-methacrylic acid) hydrogels for vascular network fabrication [91]. However, polymer fibers are less desirable in this approach when cell encapsulation is necessary because of the organic solvents such as dichloromethane needed to remove the PMMA.

For sacrificial molding in bioprinting, Miller *et al.* printed 3D rigid filament networks with carbohydrate glass as a biocompatible sacrificial template in engineered vascular networks [5]. The glass fibers were dissolved to form vessels while intervessel junctions were formed at the sites of interfilament fusions. The vessels were lined with ECs and the whole scaffold could sustain high-pressure pulsatile flow.

Compared to bioprinting directly with hydrogels, this approach provides better mechanical stiffness of vascular constructs and an easier approach to fabricating complex vascular networks.

### 4.6. Modular or Bottom-up Method

“Modular” or “bottom-up” method is to assemble predefined microscale constructs into desirable macroscale tissue constructs for the fabrication of complex constructs [1]. It has been used to pre-fabricate small units with one or more tubular structures to generate large and complex constructs. The assembly procedures can be performed manually, physically or chemically [92]. McGuigan *et al.* fabricated microscale cylindrical-shaped constructs using collagen encapsulated with cells [93]. These small units were assembled into a large tubular chamber. However, as collagen has weak mechanical properties, the modulus in this system cannot sustain high-pressure flow under perfusion. Therefore the same group modified the procedures by using gelatin crosslinked with glutaraldehyde, which performed well under perfusion [94].

**Figure 6:**
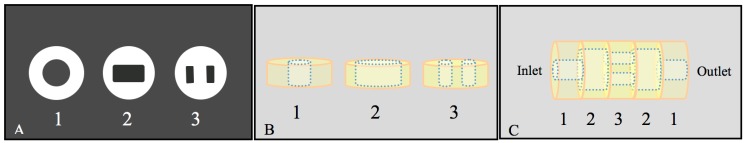
Modular method for fabricating multi-level tubular constructs. (**A**) The Mask with multi-level tubular structure for a photopatterning approach; (**B**) The microtubes created after UV-light photocuring; (**C**) Microtubes are assembled into a larger and longer tubular construct with multi-level interconnected lumens.

Du *et al.* engineered PEG constructs with multi-level vascular networks through a directed bottom-up approach. PEG microscale units with predefined internal tubular structures were created via photolithography. The units were assembled into a larger and longer tubular construct with multi-level interconnected lumens. They also encapsulated smooth muscle cells (SMCs) in the external layer and HUVECs in the internal layer of the microgel respectively, which mimicked the architecture of natural vascular networks (Figure 6) [83].

## 5. Future Perspectives

Perfusable vascular networks within hydrogels can greatly promote oxygen and nutrient transport and facilitate the investigation of cellular microenvironments and related cell behavior in hydrogels. However, before we can efficiently fabricate more complex perfusable vascular network constructs to mimic natural tissues, there still are several challenges, which need to be mastered by future research. First, since cells behave differently when attached to or encapsulated in hydrogels of different mechanical properties, these mechanical properties have to be optimized for tissue specific constructs; Second, further study is needed for improving the mechanical properties of hydrogels, which will hold complex vascular networks and sustain persistent perfusion under pulsatile pressure. Current systems for fabrication of vascular network constructs are confined to generate small and simple, parallel or lattice channels, which cannot efficiently mimic the architecture of the vascular system in the human body. Long-term perfusion will be required for the development of a mature vascularized construct; Third, some effort should be spent on mimicking the mechanical heterogeneity of native soft tissues and multilayered constructs should be created. Hydrogels with homogeneous stiffness may not be consistent with the native dynamic cell microenvironment (e.g., the multilayered vessel structures). Therefore combination of constructs with different mechanical properties and vascular structures to form a complex tissue or organ remains a challenge; Fourth, it is essential to study cell-hydrogel interactions. Understanding cell behavior and cell function in different hydrogels might help to induce individual types of cells on site and create a functional tissue construct for *in vivo* use (e.g., contraction of smooth muscle cells in hydrogels); Fifth, newly developed and optimized hydrogels and systems for bioprinting might greatly promote the development of perfusable vascular network engineering; Finally, integration of the perfusable vascular network into the human vascular system to steadily perform the expected function remains the greatest challenge of all.
